# Personal Activity Trackers and Family Engagement in a Pediatric Obesity Intervention: Randomized Controlled Trial

**DOI:** 10.2196/70341

**Published:** 2025-11-05

**Authors:** Juan Carlos Espinoza Salomon, Mahsa Babaei, Alexis Deavenport-Saman, Olga Solomon, Choo Phei Wee, Ramon Durazo-Arvizu, Abu Sikder, Payal Shah, Patricia Castillo, Larry Yin

**Affiliations:** 1 Department of Pediatrics, Ann & Robert H Lurie Children’s Hospital of Chicago, 303 E Superior St, Chicago, IL, 60611, United States, 1 312 5031499; 2 Department of Pediatrics, Northwestern University Feinberg School of Medicine, Chicago, IL, United States; 3 Department of Anesthesiology, Keck School of Medicine of the University of Southern California, Los Angeles, CA, United States; 4 Department of Pediatrics, Keck School of Medicine of the University of Southern California, Los Angeles, CA, United States; 5 Department of Pediatrics, Children’s Hospital Los Angeles, Los Angeles, CA, United States; 6 Southern California Clinical and Translational Science Institute, Los Angeles, CA, United States; 7 Biostatistics and Data Analysis Core, The Saban Research Institute, Children's Hospital Los Angeles, Los Angeles, CA, United States

**Keywords:** pediatrics, obesity, federally qualified health center, digital health intervention, underserved population

## Abstract

**Background:**

Pediatric obesity continues to be a national health crisis. Parents play a critical role in obesity interventions. Digital health interventions, such as personal activity trackers, can help better engage parents in pediatric obesity interventions and improve outcomes.

**Objective:**

This study aimed to (1) assess the feasibility and acceptability of implementing personal activity trackers as part of a comprehensive family-based lifestyle intervention for pediatric obesity (BodyWorks) in a Federally Qualified Health Center; (2) evaluate the impact of personal activity trackers on parents’ engagement, participant anthropometrics, and the overall program; and (3) examine the associations between steps per day and usage (minutes) with body composition outcomes.

**Methods:**

A total of 158 families were randomized to the control (BodyWorks) or intervention (BodyWorks + physical activity tracker) arm. Mean levels of weight-by-height outcomes, including BMI, BMI *z* scores, and BMI percent of the 95th percentile, were compared between the 2 groups.

**Results:**

There were no differences between study arms at baseline. After adjustment, there was a significant group difference in children’s BMI *z* scores from baseline to the postintervention time point (*P* for interaction=.01).

**Conclusions:**

Families in the intervention group that completed the program had slightly better weight outcomes than the controls. Engaging parents through digital health interventions may be an effective way to enhance existing pediatric obesity intervention programs.

## Introduction

Globally, 39 million children younger than 5 years of age, and 340 million between the ages of 5 and 19 years, are overweight or obese, with the prevalence of overweight and obesity reaching approximately 18% worldwide [1]. In the United States, pediatric obesity and associated comorbidities disproportionately impact low-income families and families of color [2,3]. Evidence-based pediatric obesity interventions involve multidisciplinary, multimodality, family-based therapies that are both time- and resource-intensive and may not be widely available or accessible [4].

Significant interest has emerged in leveraging digital health interventions to address pediatric obesity, including mobile apps, wearable devices, and text messaging [5]. One attractive aspect of digital health interventions is their relatively low cost to scale, particularly in low-resource settings. However, there are equity, implementation, and adoption concerns related to the growing digital divide, including gaps in access to and quality of broadband internet, access to digital devices, and the digital literacy and skills necessary to use the devices and the applications appropriately [6,7]. The COVID-19 pandemic made the digital divide especially visible when health care and educational services in the United States attempted to transition to remote delivery. The American Medical Informatics Association (AMIA) has published a statement expressing concerns that digital technology is widening health inequity generated by the digital divide rather than decreasing it [8], and the concept of digital inclusion has gained traction as a social determinant of health [9,10].

Our Federally Qualified Health Center (FQHC) uses the BodyWorks curriculum to deliver a low- to medium-intensity comprehensive behavioral family lifestyle intervention that focuses on dietary intake, physical activity, and behavioral strategies for weight loss [11]. Our team was interested in deploying digital health interventions to support the families participating in our program. Because parents play a central role in pediatric obesity management [12], we developed a parent-focused personal activity tracker (PAT) intervention. We have previously published our protocol [13] and demonstrated that our intervention reduced program attrition [14]. Here, we examined the feasibility and acceptability of implementing personal activity trackers as part of a comprehensive family-based lifestyle intervention for pediatric obesity (BodyWorks) in an FQHC. We also evaluated the impact of personal activity trackers on parents’ engagement, participant anthropometrics, and the overall program. In addition, we examined the associations between steps per day and usage (minutes) with body composition outcomes.

## Methods

### Study Design

The prospective, open-label, randomized clinical trial was conducted between 2015 and 2017 in the CHLA AltaMed General Pediatrics Clinic, a FQHC located within an academic medical center serving many underserved patients.

Participating families were enrolled if the primary child was aged 7‐18 years, had a BMI ≥85% for age and sex, and had at least one parent or caretaker able to attend the sessions with the child and willing to wear a PAT if assigned to the intervention arm. Participants were provided with incentives for their time and effort. This trial was conducted in accordance with the CONSORT (Consolidated Standards of Reporting Trials) guidelines (checklist provided in Checklist 1).

### Study Setting

Our FQHC serves as the medical home for a large, urban, medically underserved patient population of approximately 27,000 children and provides more than 100,000 patient visits annually. Approximately 75% of families identify as Latino, half of whom have limited English proficiency (LEP). Medi-Cal, California’s Medicaid program, insures 93% of patients.

### BodyWorks Curriculum

BodyWorks is the comprehensive behavioral family lifestyle intervention program, originally developed by the Office of Women’s Health of the Department of Health and Human Services. Our adaptation and implementation of the curriculum have been previously described in detail [14]. Briefly, families are referred to BodyWorks by their primary care physician. The program is offered at no cost to families. The 8-week program consists of 1 orientation and 7 classes; the groups meet weekly for 2 hours at a time. Sessions are offered in English and Spanish; families decide which session they prefer. Each session begins with all participants together for physical activity and a healthy snack. Parents and children are then separated to receive tailored curricula from a multidisciplinary team. The curriculum focuses on nutrition, physical activity, stress, sleep, body image, bullying, the role of the media, and how to continue lessons learned.

### Study Procedures and Data Collection

#### Anthropometrics

Height and weight were measured weekly for children. BMI, BMI percentile for age and sex, and BMI percent of the 95th percentile (%BMIp95) were all calculated using standard approaches from weekly measurements. We used the Centers for Disease Control and Prevention (CDC) definitions of overweight and obesity in childhood [15].

#### Surveys

Enrolled families completed a demographic survey on the first day of BodyWorks. At completion, all families completed a technology use survey, which consisted of questions such as, “Do you own a smartphone?” “Do you use text messaging, email, or any other social platform?” and “Would you like to receive BodyWorks communication through digital health technology?” Finally, families in the intervention arm completed an exit survey about their experience using PATs, consisting of six 5-point Likert scale questions.

#### Randomization

To ensure balanced allocation of language groups and prevent cross-contamination, block randomization was used to assign whole groups to either arm of the study. The primary aim for program completion was defined as a family attending at least 4 of the 7 sessions.

#### Intervention Arm (BodyWorks + PAT)

Participants were assigned to either the intervention arm or the control arm. In the intervention arm, the parent and the child aged ≥13 years received the PAT, Fitbit (Fitbit). BodyWorks staff instructed the intervention arm participants on PAT use, including charging and usage practices. They were instructed to wear PAT devices on the wrist of the nondominant arm for a minimum of 20 hours per day. All participants received a US $15 gift card for agreeing to participate. Those randomized to receive a PAT received an additional US $50 gift card for returning the device at the end of the trial.

#### Control Arm (BodyWorks Only)

Participants in the control arm attended the same sessions and received the same curriculum as those in the intervention.

#### Outcomes

Program completion, PAT exit survey (attitudes and perceptions), BMI, BMI *z* score, BMI percentile, %BMIp95, number of steps per day, and usage time per day.

#### Covariates

Demographic characteristics, session attendance, and technology access and preferences.

### Statistical Analysis

Descriptive statistics were used to summarize and describe the distribution of demographic variables. Continuous variables were summarized as means and SDs, whereas percentages were used for categorical variables. We examined family and child demographic differences between the intervention and control groups by conducting chi-square tests or Fisher exact tests, and differences in age and relative weight outcomes by conducting 2-sample *t* tests.

For the primary study outcomes, a generalized estimating equation approach was conducted to examine changes in BMI *z* scores and %BMIp95 between children in the intervention and control groups, adjusting for sociodemographic and program characteristics. The nonadditive effects of children in the intervention compared to the control groups were also examined (eg, whether the change from baseline to follow-up differed between children in the intervention and control groups) by including intervention group-by-visit interaction terms. Covariates included age, sex, LEP, household income as a percentage of the federal poverty level, and program completion. A multivariate linear regression model assessed relationships between child demographics and parent and child total steps per day, and minutes of PAT use daily, while adjusting for the percent of days with valid data. The effect of total steps per day and PAT usage on change in relative weight measures (BMI *z* score and BMI percent of 95th percentile) was estimated using a generalized estimating equation model, adjusting for baseline levels of relative weight measure, ethnicity, household size, gender, household income, and percent days with valid data. The significance level was set at 5% with 2-sided tests. All statistical computations were done in Stata/SE 18 (StataCorp).

### PAT Data Extraction

PAT data were extracted for each participant through the web application programming interface (API) using a customized Python script. A developer app account was registered through the API portal and configured for OAuth 2.0 and client-side authentication. After running the Python script, OAuth 2.0 authentication occurred through a web browser, where each user’s PAT login credentials were used, and all data types were selected for sharing. After authentication, an access token was retrieved from the Representational State Transfer API call to grant authorization using a unique user ID and client secret code assigned to the PAT developer app. The access token was then used for a customized Representational State Transfer API call to retrieve steps, distance, and minutes, including sedentary, lightly active, fairly active, and very active steps from 2015 to 2020. The extracted JavaScript Object Notation data were parsed and formatted into a CSV file.

### PAT Data Aggregation

Each CSV file contained all available data for a specific PAT account from the program’s inception to its conclusion. A Python script was used to aggregate all the individual data into a master CSV file. Since PAT accounts were reused, participants were mapped to cycles for tracking purposes. In addition, a mapping of each cycle to the study timeframe was used to filter and organize data in the master CSV file. The final dataset was organized into cycles and participants with data points such as date, day of week, steps, active minutes, and distance traveled.

### Ethical Considerations

The Children’s Hospital Los Angeles Institutional Review Board approved the study (CHLA-15-00269), and all participants signed consent or assent in their preferred language. To protect participant privacy and confidentiality, only data necessary to meet the study objectives were collected. Personal identifiers were removed and replaced with coded study identification numbers, and data were stored in password-protected, secure institutional databases. Study participants received a US $15 gift card and an additional US $50 gift card upon returning their PATs.

## Results

### Study Enrollment and Completion

**Figure 1.**
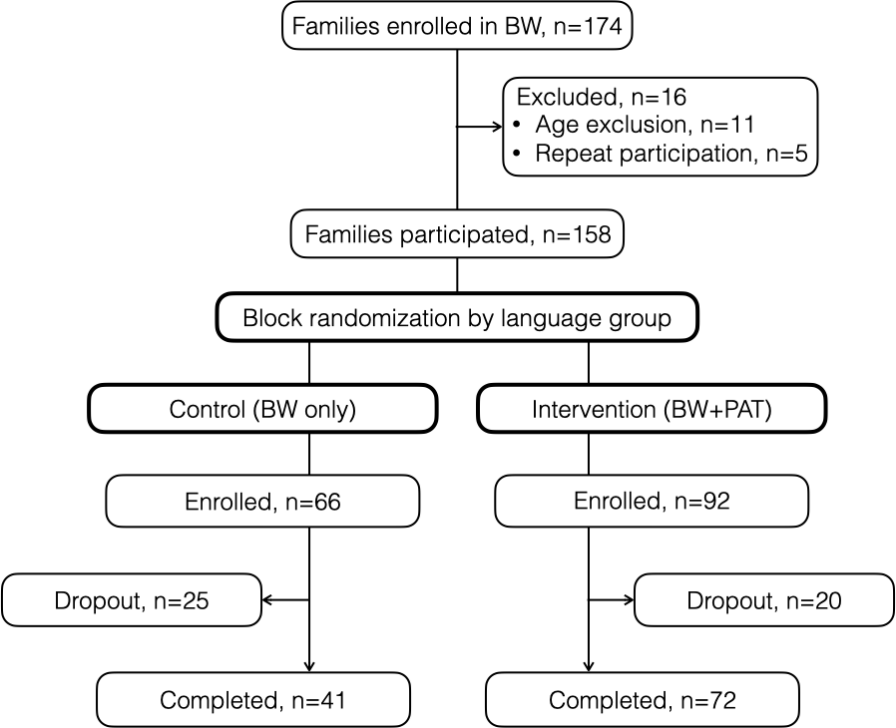
CONSORT (Consolidated Standards of Reporting Trials) diagram for the BodyWorks study. BW: BodyWorks; PAT: personal activity tracker.

Initially, 174 families participated in BodyWorks; however, 11 families were excluded from the analysis, as their only participating child was younger than 7 years of age. An additional 5 families were excluded because it was their second or third time in the BodyWorks program. The study sample comprised 158 families, including 200 children, with 80 children in the control group and 120 in the intervention group (Figure 1).

### Sociodemographic and Anthropometric Characteristics

#### Family-Level Characteristics

The demographics of BodyWorks participants represented the FQHC from which they were recruited and the local community (Table 1). Most families were Latino, had LEP, had household incomes below 200% of the federal poverty level, and had household sizes of 5 or more people. There were no significant differences between the intervention and control groups at baseline.

**Table 1.** Baseline sociodemographic characteristics of families and children by intervention and control groups.

Characteristic and study arm	Control	Intervention	Total	*P* value
Families				
Ethnicity, n/N (%)				.72
Hispanic Latino	60/66 (93.8)	88/92 (95.7)	148/158 (94.9)	
Not Hispanic or Latino	4/66 (6.2)	4/92 (4.3)	8/158 (5.1)	
Household Income as % FPL^a^, n/N (%)				≥.99
<200% FPL	50/66 (92.6)	77/92 (92.8)	127/158 (92.7)	
>200% FPL	4/66 (7.4)	6/92 (7.2)	10/158 (7.3)	
Household size, n/N (%)				≥.99
5+	34/66 (54)	47/92 (52.8)	81/158 (53.3)	
1-4	29/66 (46)	42/92 (47.2)	71/158 (46.7)	
Limited English proficiency, n/N (%)				.05
Yes	40/66 (62.5)	71/92 (77.2)	111/158 (71.2)	
No	24/66 (37.5)	21/92 (22.8)	45/158 (28.8)	
Children				
Sex, n/N (%)				.77
Female	34/80 (42.5)	54/120 (45)	88/200 (44)	
Male	46/80 (57.5)	66/120 (55)	112/200 (56)	
Age (years), mean (SD)	10.3 (1.8)	11.3 (2.8)	10.9 (2.5)	.04
Height (cm), mean (SD)	143.4 (13.8)	145.1 (13.5)	144.4 (13.6)	.33
Weight (kg), mean (SD)	58.7 (21.7)	62.5 (24.2)	61.0 (23.3)	.31
BMI, mean (SD)	27.7 (5.5)	28.7 (6.9)	28.3 (6.4)	.45
BMI percentile, mean (SD)	97.6 (2.2)	97.1 (3.2)	97.3 (2.9)	.70
BMI *z* score, mean (SD)	2.1 (0.4)	2.1 (0.5)	2.1 (0.4)	.60
%BMIp95^b^, mean (SD)	120% (20%)	120% (20%)	120% (20%)	.56

a FPL: federal poverty level.

b %BMIp95: BMI percent of the 95th percentile.

#### Child-Level

There was a significant age difference (*P*=.04) between children in the intervention and control groups (Table 1). At baseline, there were no differences in BMI *z* scores, BMI percentiles, or % BMI95 between the intervention and control groups. There were also no other significant differences between children in the 2 groups at baseline.

### Technology Use

A total of 132 families completed the technology use survey. Of these, 130 (98.5%) respondents reported having a cell phone, with 39 (30.2%) having iOS (Apple) devices and 83 (64.3%) having Android (Google) devices. Almost all families (127/132, 96%) used their phone for texting, with fewer families using their phones for social media (99/132, 75%) and email (96/132, 73%). About 86% (113/132) of families had an unlimited data plan.

### Weight Outcomes

#### Unadjusted Weight Outcomes

The weight outcomes are presented in Table 2 among all children who enrolled in the program and those who completed the intervention. Among all children, there was a significant difference in the change of BMI *z* scores between children in the control and intervention groups (mean –0.00, SD 0.07 vs mean –0.04, SD 0.11; *P*=.02; Table 2). There were, however, no differences in the change of %BMIp95 between children in the control and intervention groups (mean 121.6%, SD 19.8% vs mean 119.7%, SD 22.2%; *P*=.56). Among children who completed the intervention, there were no differences in the change in BMI *z* scores between children in the control and intervention groups (mean –0.01, SD 0.07 vs mean –0.04, SD 0.12; *P*=.10; Table 2). There were also no differences in the change in %BMIp95 between children in the control and intervention groups (mean 121%, SD 21.7% vs mean 118.7%, SD 20.9%; *P*=.53).

**Table 2.** Summary statistics for children’s body composition measures by intervention group: enrolled and completed.

Characteristic and study arm	Control	Intervention	Total	*P* value
Enrolled in a study				
Sex, n/N (%)				.77
Female	34/80 (42.5)	54/120 (45)	88/200 (44)	
Male	46/80 (57.5)	66/120 (55)	112/200 (56)	
Height (cm) at baseline, mean (SD)	143.37 (13.85)	145.12 (13.47)	144.41 (13.62)	.38
Weight (kg) baseline, mean (SD)	58.72 (21.71)	62.51 (24.24)	60.99 (23.28)	.26
BMI at baseline, mean (SD)	27.69 (5.46)	28.75 (6.91)	28.32 (6.38)	.25
BMI percentile at baseline, mean (SD)	97.61 (2.20)	97.09 (3.25)	97.30 (2.88)	.21
BMI *z* score at baseline, mean (SD)	2.141(0.38)	2.10 (0.45)	2.12 (0.43)	.52
%BMIp95 at baseline, mean (SD)	122.1% (20.9%)	121.1% (23%)	121.5% (22.1%)	.77
Height (cm) at follow-up, mean (SD)	144.35 (13.69)	145.66 (13.24)	145.17 (13.38)	.53
Weight (kg) at follow-up, mean (SD)	59.38 (22.30)	61.80 (22.64)	60.90 (22.48)	.49
BMI at follow-up, mean (SD)	27.61 (5.42)	28.29 (6.44)	28.04 (6.07)	.47
BMI percentile at follow-up, mean (SD)	97.64 (2.14)	96.54 (4.23)	96.95 (3.63)	.05
BMI *z* score at follow-up, mean (SD)	2.15 (0.36)	2.07 (0.48)	2.10 (0.44)	.23
%BMIp95 at follow-up, mean (SD)	121.6% (19.8%)	119.7% (22.2%)	120.4% (21.3%)	.56
BMI *z* score difference, mean (SD)	–0.00 (0.07)	–0.04 (0.11)	–0.02 (0.10)	.02
%BMIp95 difference, mean (SD)	–0.1% (3.7)	–1.1% (3.3)	–0.7% (3.5)	.07
BMI percentile difference, mean (SD)	–0.10 (0.71)	–0.56 (1.78)	–0.39 (1.49)	.04
Completed study				
Sex, n/N (%)				.60
Female	20/50 (40)	43/95 (45.3)	63/145 (43.4)	
Male	30/50 (60)	52/95 (54.7)	82/145 (56.6)	
Height (cm) at baseline, mean (SD)	145.49 (14.23)	144.37 (13.92)	144.76 (13.99)	.65
Weight (kg) baseline, mean (SD)	60.94 (24.73)	61.00 (22.44)	60.98 (23.17)	.99
BMI at baseline, mean (SD)	27.76 (6.13)	28.35 (6.20)	28.15 (6.16)	.59
BMI percentile at baseline, mean (SD)	97.46 (2.24)	97.26 (2.70)	97.33 (2.55)	.66
BMI *z* score at baseline, mean (SD)	2.11 (0.39)	2.09 (0.40)	2.10 (0.40)	.79
%BMIp95 at baseline, mean (SD)	121% (21.9%)	120% (20.6%)	120.3% (21%)	.78
Height (cm) at follow-up, mean (SD)	146.43 (14.19)	145.40 (13.57)	145.75 (13.74)	.67
Weight (kg) at follow-up, mean (SD)	61.71 (24.94)	61.11 (22.31)	61.32 (23.15)	.88
BMI at follow-up, mean (SD)	27.74 (6.16)	28.03 (6.25)	27.93 (6.20)	.79
BMI percentile at follow-up, mean (SD)	97.39 (2.37)	96.64 (3.93)	96.89 (3.49)	.22
BMI *z* score at follow-up, mean (SD)	2.12 (0.38)	2.05 (0.45)	2.07 (0.43)	.40
%BMIp95 at follow-up, mean (SD)	121% (21.7%)	118.7% (20.9%)	119.5% (21.1%)	.53
BMI *z* score difference, mean (SD)	–1.2% (6.8%)	–4.2% (11.5%)	–3.2% (10.3%)	.10
%BMIp95 difference, mean (SD)	–0.01 (0.04)	–0.01 (0.04)	–0.01 (0.04)	.24
BMI percentile difference, mean (SD)	–0.20 (0.77)	–0.63 (1.91)	–0.48 (1.63)	.13

#### Adjusted Weight Outcomes

The adjusted multivariable regression demonstrated a significant difference in BMI *z* scores between children in the intervention and control groups from baseline to the postintervention time point (β=–.035; *P* value for interaction=.01; Table 3). There were no statistically significant differences in %BMIp95 between children in the intervention and control groups from baseline to the postintervention time point (β=–.008; *P* value for interaction=.19). Changes in BMI percentile from baseline differed between intervention groups (β=–.571; *P* value for interaction <.01). All models were adjusted for baseline BMI, age-gender–specific relative weight measure, age in years, household income, LEP, gender, and study completion.

The relative weight change in the control group is 0.003, –0.001, and –0.023 for BMI *z* scores, %BMIp95, and BMI percentile, respectively. In the intervention group, the corresponding change is 0.003–0.035= –0.032, –0.001–0.008=–0.009, and –0.023–0.571=–0.594. The interaction terms, which represent the change difference between intervention and control groups, are –0.035 (*P*=.01), ‐0.008 (*P*=.19), and 0.571 (*P*=.004) for BMI *z* scores, %BMIp95, and BMI percentile. In summary, changes in relative weight measures in the control group are not statistically significant, as seen from the 95% CIs, which include 0. In contrast, a statistically significant decrease in the average of the 3 measures of weight-by-height was observed in the intervention group.

**Table 3.** Multivariable generalized estimating equation (GEE) model assessing the change in relative weight measures by intervention arm, adjusting for baseline levels, age, household income, limited English proficiency, gender, and study completion.

Variable	BMI *z* score		%BMIp95^a^		BMI percentile	
	β (95% CI)	*P* value	β (95% CI)	*P* value	β (95% CI)	*P* value
Intercept	–0.020 (–0.06 to 0.02)	.32	0.008 (–0.01 to 0.02)	.35	-8.12 (–14.82 to –1.42)	.02
Baseline relative weight	1.012 (1.00 to 1.04)	<.01	0.998 (1.00 to 1.01)	<.01	1.09 (1.02 to 1.16)	<.01
Study arm						
Intervention	0.001 (–0.00 to 0.01)	.76	0.000 (–0.001 to 0.002)	.62	0.00 (–0.10 to 0.11)	.96
Time						
Postintervention	0.003 (–0.01 to 0.02)	.72	–0.001 (–0.01 to 0.01)	.83	–0.02 (–0.13 to 0.09)	.68
Study arm x time						
Intervention x postintervention	–0.035 (–0.06 to –0.01)	<.01	–0.008 (–0.02 to 0.00)	.19	–0.57 (–0.96 to –0.18)	<.01
Age (years)						
≥13	0.007 (–0.01 to 0.02)	.3	0.001 (–0.00 to 0.01)	.65	0.24 (0.04 to 0.45)	.02
Household income (% FPL^b^)						
>200% FPL	0.000 (–0.02 to 0.02)	≥.99	–0.001 (–0.01 to 0.01)	.8	–0.01 (–0.31 to 0.30)	.97
Limited English proficiency						
No	–0.007 (–0.02 to 0.02)	.37	0.000 (–0.01 to 0.01)	.92	–0.23 (–0.54 to 0.07)	.14
Sex (child)						
Male	–0.011 (–0.03 to 0.00)	.12	0.002 (–0.01 to 0.00)	.47	–0.23 (–0.45 to –0.01)	.04
Completed study						
Yes	–0.013 (–0.02 to –0.003)	.01	–0.006 (–0.01 to –0.001)	.01	–0.12 (–0.27 to 0.03)	.12

a %BMIp95: BMI percent of the 95th percentile.

b FPL: federal poverty level.

### Personal Activity Tracker Data

A total of 146 parents were assigned PATs, generating 6826 data points over 43 to 52 days each. Of these, 3662 (53.7%) were valid or nonzero data points. Almost two-thirds (63%) of participants had at least 50% valid data. Over a week, participants were most likely to generate data on Wednesdays and least likely on Sundays.

Excluding days with no data, the mean (SD) step count per user per day was 8418 (8186). The mean (SD) minutes of physical activity per user per day was 244.7 (289.7). The mean (SD) miles traveled per user daily was 3.6 (3.53). Participants were most active on Thursdays (9364 daily steps) and Fridays (8917 daily steps), and least active on Saturdays (7651 daily steps) and Sundays (6740 daily steps).

Table 4 shows the multivariable linear regression models for the 2 outcome measures recorded in the PAT instrument, including the number of daily steps and usage (minutes) for parents and children. The resulting 4 models included 4 demographic variables: household size, percent, gender, household income, and ethnicity. The models were adjusted for percent days with valid data and the number of sessions attended. Parents living in households with 1 to 4 people had an average of 2907 fewer steps per day compared to those in households with 5+ individuals (β=–2907; *P*=.02), as well as 60 minutes less (β=–60; *P*=.05) of usage time. Fathers had an average of 3270 fewer steps than mothers (β= –3270; *P*=.07). Percent days with valid data were significantly associated with steps per day and minutes of use among parents and children, whereas the number of sessions attended did not impact either outcome measure.

In Table 5, an increase of 1000 steps per day resulted in an increase in BMI *z* score, %BMI95, and BMI percentile from baseline of 0.009 (*P* value for interaction ≤.01), 0.004 (*P*≤.01), and 0.07 (*P*=.05), respectively.

A 30-minute increase in PAT usage was associated with an increase from baseline of BMI *z* score, %BMI95, and BMI percentile of 0.009 (*P*=.03), 0.004 (*P*=.03), and 0.054 (*P*=.05; Table 6).

**Table 4.** Multivariable linear regression for the number of steps per day and usage (minutes) among parents and children.

Variable	Parent				Child			
	Steps/day		Minutes/day		Steps/day		Minutes/day	
	β	*P* value	β	*P* value	β	*P* value	β	*P* value
Intercept	–594	.76	39	.47	3215	.25	142	.06
Household size								
1 to 4	–439	.55	5	.80	–2907	.02	–60	.05
Percent days with valid data	1441	<.01	35	<.01	939	.04	26	.03
Sex								
Male	–3270	.07	–54	.28	–1787	.13	–50	.10
Household income (% FPL^a^)								
>200% FPL	–2309	.11	–53	.18	–1991	.28	–54	.27
Ethnicity								
Hispanic or Latino	1382	.38	–29	.51	2245	.32	20	.73
Number of sessions attended	600	.10	21	.03	213	.60	0	.98

a FPL: federal poverty level.

**Table 5.** Child body composition changes and parent FitBit steps. The rate of change of BMI *z* score, %BMI over 95th percentile, and BMI percentile increases with increasing activity, as measured by the number of steps. That is, for BMI *z* score, %BMI over 95th percentile, BMI percentile, the rate of change increases per 1000 steps increase. Estimates of the rate of change are adjusted for the baseline outcome, race/ethnicity, household size, percent days with valid data, sex, and household income.

Variable	BMI *z* score		BMI % over 95 percentile		BMI percentile	
	Effect (95% CI)	*P* value	Effect (95% CI)	*P* value	Effect (95% CI)	*P* value
Intercept	–0.026 (–0.087 to 0.035)	.40	–0.017 (–0.042 to 0.008)	.18	2.334 (0.991 to 5.659)	.17
Steps/day	–0.002 (–0.008 to 0.003)	.41	–0.002 (–0.003 to 0.000)	.10	0.045 (0.006 to 0.096)	.08
Visit						
Follow-up	–0.072 (–0.127 to –0.017)	.01	–0.028 (–0.049 to –0.007)	.01	0.584 (–1.181 to 0.013)	.06
Time × steps/day						
Follow-up	0.009 (0.003 to 0.016)	<.01	0.004 (0.001 to 0.006)	<.01	0.070 (0.001 to 0.140)	.05
Baseline relative weight	1.010 (0.964 to 1.056)	<.01	1.017 (0.993 to 1.040)	<.01	0.972 (0.935 to 1.009)	<.01
Ethnicity						
Hispanic or Latino	0.025 (0.008 to 0.042)	<.01	0.008 (0.001 to 0.016)	.02	0.102 (–0.007 to 0.210)	.07
Household size						
1 to 4	–0.008 (0.008 to 0.042)	.24	–0.005 (–0.014 to 0.003)	.21	0.032 (–0.199 to 0.134)	.70
Percent days with valid data	0.003 (–0.012 to 0.017)	.72	0.002 (–0.001 to 0.006)	.20	0.116 (–0.233 to 0.001)	.05
Sex						
Male	–0.010 (–0.065 to 0.045)	.73	–0.007 (–0.018 to 0.004)	.22	0.486 (0.061 to 0.912)	.03
Household Income (% FPL^a^)						
>200% FPL	–0.022 (–0.085 to 0.040)	.49	–0.013 (–0.035 to 0.008)	.22	0.395 (–0.212 to 1.002)	.20

a FPL: federal poverty level.

**Table 6.** Child body composition changes and parent FitBit use in minutes. Estimates of the rate of change are adjusted for the baseline outcome, race or ethnicity, household size, percent days with valid data, sex, and household income.

Variable	BMI *z* score		BMI % over 95 percentile		BMI percentile	
	Effect (95% CI)	*P* value	Effect (95% CI)	*P* value	Effect (95% CI)	*P* value
Intercept	–0.023 (–0.071 to 0.025)	.35	–0.015 (–0.037 to 0.006)	.17	–0.016 (–3.824 to 3.792)	.99
Minutes/day	–0.004 (–0.009 to 0.001)	.09	–0.002 (–0.004 to 0.000)	.03	0.000 (–0.054 to 0.054)	.99
Time						
Follow-up	–0.066 (–0.127 to –0.005)	.03	–0.027 (–0.050 to –0.004)	.02	–0.439 (–0.904 to 0.027)	.07
Time × minutes/day						
Follow-up	0.009 (0.001 to 0.018)	.03	0.004 (0.000 to 0.007)	.03	0.054 (–0.001 to 0.108)	.05
Baseline relative weight	1.010 (0.982 to 1.038)	<.01	1.016 (0.996 to 1.036)	<.01	0.999 (0.959 to 1.039)	<.01
Ethnicity						
Hispanic or Latino	0.030 (0.018 to 0.042)	<.01	0.010 (0.003 to 0.016)	<.01	0.185 (0.123 to 0.247)	<.01
Household size						
1 to 4	–0.014 (–0.027 to –0.002)	.03	–0.007 (–0.014 to –0.001)	.03	–0.119 (–0.285 to 0.047)	.16
Percent days with data	0.005 (–0.005 to 0.014)	.35	0.003 (–0.000 to 0.006)	.08	–0.036 (–0.144 to 0.073)	.52
Sex						
Male	–0.012 (–0.042 to 0.018)	.44	–0.007 (–0.017 to 0.003)	.16	0.144 (–0.169 to 0.457)	.37
Household income (% FPL^a^)						
>200% FPL	–0.025 (–0.078 to 0.029)	.36	–0.014	.19	0.024 (–0.386 to 0.433)	.91

a FDL: federal poverty level.

### PAT Exit Survey

A total of 83 participants completed the PAT exit survey. The survey included six 5-point Likert scale items to assess (1) PAT comfort, (2) ease of charging, (3) enjoyment, (4) motivation to do more activity, (5) utility of weekly reports and feedback, and (6) consideration of PAT purchase in the future. The responses were overwhelmingly positive, with ≥95% of respondents agreeing or strongly agreeing with every statement.

## Discussion

### Principal Findings

In this randomized control trial, we demonstrated the feasibility of using a digital health intervention involving PATs to positively impact a predominantly low-income, Latino patient population participating in a pediatric obesity intervention at an FQHC. Families reported that their PATs were easy to use, comfortable to wear, and motivating. This is consistent with other studies in the literature [16,17]. In addition, there was a significant decrease in BMI *z* scores among children in the intervention compared to children in the control group from baseline to the postintervention time point. Interestingly, within the intervention arm, an increase of 1000 steps per day and a 30-minute increase in PAT usage resulted in an increase in BMI *z* score, %BMI95, and BMI percentile from baseline to the postintervention time point. Given that this group overall had improved weight outcomes, the apparently paradoxical weight increase among those with higher physical activity may be related to an increase in muscle mass in the short term as the family became more active.

Digital health interventions have the potential to be powerful adjuncts to existing programs and interventions. Low-cost technologies can scale rapidly to provide more and better care to more patients. In the past, there have been concerns about the ability to meaningfully deploy technology-based interventions in low-income, non–English-speaking, or otherwise underserved communities. This has been justified with concerns about access to or comfort with mobile technologies [7]. While national surveys do indicate a gap in access to technologies such as smartphones for the poorest Americans, this may be a result of their methodology and sampled population [18]. Our study and others have consistently demonstrated that, at least among urban communities, access to smartphones is nearly ubiquitous, regardless of socioeconomic status, except at the very extremes of poverty. Out of the 132 families who completed our technology survey, 94% reported owning a smartphone and using it regularly for texting, social media, and email. This finding underlines the importance of understanding not only the needs, but also the strengths and resources of any community for which an intervention is planned.

With regard to PAT use, most participants were able to generate valid data from 50% or more of their days in the study. Common barriers for not generating valid data included forgetting to wear the PAT or not charging the PAT. Wednesdays were the day with the most valid data; this may be related to the fact that the BodyWorks program occurred on Monday, Tuesday, and Wednesday nights, which provided a natural reminder for patients to charge and wear their PAT. Similarly, families were most active on Thursdays and Fridays, and least active over the weekend. This may be due to the effect of being reminded during the Monday-Wednesday sessions, and families become more active in the following days. It is interesting to note that the data shows the least amount of activity over the weekend. This may reflect families genuinely being less active or possibly forgetting to wear or charge their PATs over the weekend.

The BodyWorks program has made several accommodations and adaptations to meet the needs of our patient population and encourage families to participate and engage. Because childcare can be costly, all children in a family are welcome to join the group and participate, regardless of age or weight status. For parents who work or are otherwise unable to attend themselves, other family members (typically grandparents or adult siblings) may participate in their place. The entire program is supported by a bilingual team that is able to provide culturally and linguistically competent care to all families. In 2020, when the program went online in response to the COVID-19 pandemic, we continued to offer Spanish- and English-language online groups, and families could connect with a program coordinator if they needed help familiarizing themselves with the teleconferencing platform used.

From a theoretical standpoint, personal activity trackers enable self-monitoring, a well-established behavior change technique that has been shown to increase physical activity in adults [19-21]. In our study, we further supported behavior change by providing coaching, another well-established behavior change technique. Each week, a member of the study team reviewed each participant’s data with them, praising achievements, identifying barriers to physical activity, and discussing solutions. This created an opportunity for personalized attention for each participant.

The present research demonstrated a statistically significant improvement in weight outcomes for children in the intervention arm from baseline to the postintervention time point compared to those in the control arm. However, these changes are unlikely to be clinically significant. Existing clinical guidelines recommend 5%‐10% weight loss for adults and 4%‐7% change in BMI percentile or *z* score for children to begin to see metabolic benefit [21,22]. Neither of these thresholds was achieved by our cohort. Furthermore, we only report on weight outcomes at 8 weeks, a relatively short period of time. Future studies will need to evaluate the long-term impact of this intervention, as the durability of interventions can vary significantly from their short-term impact. Finally, it is worth remembering that while the goal of most adult obesity interventions is weight loss, the goal of pediatric obesity interventions varies by age, obesity severity, comorbidities, and developmental stage. Weight maintenance, decreasing growth velocity, or weight loss may all be appropriate goals depending on the patient [20].

One interesting aspect of our study is that, despite being a pediatric obesity program, our digital health intervention targeted parents. Parental education and behavior change are the critical drivers of weight management in pediatric obesity, particularly among younger children [22]. Parent-only interventions have been shown to be effective modalities for pediatric obesity [23,24]. Engaging parents in making changes in the home and adopting healthier habits leads to children adopting healthier lifestyles and, over time, improved weight outcomes.

There were departures from the original study protocol. For example, each family was supposed to complete paper-based activity logs each week. These were intended to serve as a control for the personal activity trackers. However, from the beginning of the study, we observed very poor completion rates, particularly among the families who had PATs. One participant asked, “We look at my Fitbit every week, why do I have to fill this out?” After 2 cycles, the study team decided to no longer collect physical activity logs. While this may have impacted our overall analysis and the opportunity to compare self-reported physical activity with physical activity captured by the personal activity trackers, it was felt to be the right decision for the families participating in our program. In the original protocol, only one parent was to receive a personal activity tracker. Within the first 2 cycles, it became clear that for families with 2 parents participating in BodyWorks, determining which parent received the PAT was sometimes a source of conflict. Therefore, we decided to allow both parents to have a PAT if they were both interested in using one.

### Strengths and Limitations

This study has several strengths. A large number of families participated, allowing for a robust statistical analysis. Our overall intervention design is straightforward and low-cost and could be replicated in clinical settings with minimal expenses. While PATs can be expensive, most modern smartphones are now capable of tracking activity, allowing this study to be replicated without the need to purchase any additional devices. Having demonstrated success in a relatively low-resource setting, we anticipate that this approach would be at least as successful in higher-resource settings. While encouraging, there are also several important limitations. The participants were relatively homogenous, being predominantly Latino, low-income, and having LEP. This may limit the generalizability of our findings to other settings. There is an inherent self-selection bias in our study population, as we recruited families who had already agreed to attend a structured obesity intervention program. These families likely had a higher baseline level of motivation, which may have impacted our findings and raises the question of whether other, less motivated populations would have similar outcomes. This analysis only evaluated short-term (2 months) weight outcomes; the durability of the impact remains to be evaluated.

### Conclusions

Leveraging PATs to enhance an existing pediatric obesity intervention in an FQHC is feasible and may improve weight outcomes. Family income and English proficiency did not impact participants’ ability to benefit from the technology. Future studies should further evaluate the real-world effectiveness and long-term impact of this approach.

## Supplementary material

10.2196/70341 Checklist 1 CONSORT-EHEALTH (V 1.6.1) checklist.

## Abbreviations

%BMIp95: BMI percent of the 95th percentile
AMIA: American Medical Informatics Association
API: application programming interface
CDC: Centers for Disease Control and Prevention
CONSORT: Consolidated Standards of Reporting Trials
FQHC: Federally Qualified Health Center
LEP: limited English proficiency
PAT: personal activity tracker
